# Thrombolytic therapy during resuscitation for pulmonary embolism-related out-of-hospital cardiac arrest: perhaps not the ideal solution for everyone

**DOI:** 10.1186/s13054-020-2803-0

**Published:** 2020-02-24

**Authors:** Patrick M. Honore, Cristina David, Aude Mugisha, Rachid Attou, Sebastien Redant, Andrea Gallerani, David De Bels

**Affiliations:** ICU Department, Centre Hospitalier Universitaire Brugmann-Brugmann University Hospital, Place Van Gehuchtenplein, 4, 1020 Brussels, Belgium

Javaudin et al. [1] recommended that for cases of out-of-hospital cardiac arrest (OHCA) for which a cause is not obvious, pulmonary embolism (PE) should be suspected if the initial rhythm is nonshockable and there is a history of thromboembolism (TE). In accordance with the guidelines of the American Heart Association, these patients could be treated with systemic thrombolysis (ST) during resuscitation (low level of evidence) [1]. We would like to add some comments. First, recent studies have shown that ultrasound-facilitated catheter fibrinolysis relieves right ventricular pressure overload with a lower risk of major bleeding and intracranial hemorrhage than historical rates with ST [2]. However, further research is required to determine the optimal application of this technique in the setting of acute PE [2]. Second, the insertion of an emergency veno-arterial extracorporeal membrane oxygenation (VA-ECMO) catheter should be considered before starting ST. VA-ECMO can be a lifesaving therapeutic consideration, either as an adjunct to definitive management strategies (surgical/catheter embolectomy, thrombolysis) or on its own [3]. According to a recent systematic review, VA-ECMO for selected patients with massive PE is associated with good outcome [3].

Third, after failure of thrombolysis, surgical embolectomy or catheter embolectomy should be considered in selected centers [3]. Fourth, published cases of thrombolysis for massive PE during pregnancy and the postpartum period suggest acceptable maternal and fetal survival even with CA [4]. In the postpartum period, given the high risk of major bleeding with thrombolysis, other therapeutic options (catheter or surgical thrombectomy, VA-ECMO) should be considered if available [4]. Lastly, chronic thromboembolic pulmonary hypertension (CTEPH) is a pulmonary vascular disease caused by chronic obstruction of major pulmonary arteries and often occurs after an initial PE or TE [5]. The authors note the importance of a past history of PE or TE as a risk factor and should therefore consider CTEPH as well. CTEPH can be cured by pulmonary endarterectomy (PEA), a challenging procedure for which patient selection and perioperative management are complex, requiring significant experience [5]. We had a 45-year-old patient with CTEPH who, after failed thrombolysis, was transferred to another center for PEA and achieved a full recovery [5]. Thrombolysis may not be the cure for everyone. A clear step by step approach should be considered in case of failed thrombolysis.

## Data Availability

Not applicable.

## Abbreviations

OHCA: Out-of-hospital cardiac arrest
PE: Pulmonary embolism
CA: Cardiac arrest
TE: Thromboembolism
ST: Systemic thrombolysis
VA-ECMO: Veno-arterial extracorporeal membrane oxygenation
CTEPH: Chronic thromboembolic pulmonary hypertension
PEA: Pulmonary endarterectomy
